# The Report of the City Coroner

**Published:** 1910-03-26

**Authors:** 


### THE REPORT OF THE CITY CORONER.

The annual report of the City Coroner deals with several interesting points which urgently need consideration. During the past year Dr. Waldo has held 422 public inquests, including non-fatal fire inquests, in which the causes of death were apportioned as follows: 143 natural causes; 276 unnatural, or "evidence insufficient to show whether natural or otherwise"; while in 22 open verdicts were returned. Of the 276 deaths from other than natural causes, 196 were due to accident, and 36 to criminal acts. In 27 cases the jury returned verdicts of "self-destruction while insane," and in nine cases of "*felo-de-se.*" Dr. Waldo points out that this latter verdict has been abolished in Australia, and it is time that it was abolished in England. As a result of the inquests held during the year many suggestions of a useful and far-reaching character have been made by juries, and it is only fair to the City Coroner to say that the genesis of such expressions in favour of much needed reforms lay in his own excellent summing-up in the majority of cases. The City owes a debt of gratitude to Dr. Waldo for his persistent energy in calling attention to certain matters; the indebtedness of the medical profession, we venture to think, is scarcely less. Prompted by him, juries have dealt with such practical questions as the preservation of bodies pending examination, the necessity of enforcing the Cleansing of the Persons Act, the strengthening of certain sections of the Municipal Corporations Act, protection of infant life, and the Midwives Act—suggesting in regard to the last, for instance, that the payment of fees be made compulsory by the authorities to doctors where the latter are called in by the midwife. As a result, it is satisfactory to note that in one matter at least the jury's recommendations have been carried out. The City mortuary now possesses a Herscher's formalin apparatus for the preservation of dead bodies, and is the only mortuary in connection with a Coroner's Court in this country which can boast of such an installation. This apparatus has already proved of service in the actual preservation, leading to the subsequent identification, of dead bodies, especially in one case, where a body was kept for four months before it was identified. The sanitary and hygienic arguments in favour of attaching such preservation chambers to every public mortuary need no detailing here, and the coroners' juries have done well to urge the establishment of refrigerating mortuary accommodation upon the County Council. The fact that the latter has not seen fit to move in the matter is certainly not due to any want of energy on the part of the City Coroner, who has not hesitated to back up the recommendations of his various juries as far as was possible. One section of Dr. Waldo's report touches an important subject—fires and their prevention. During the year he has inquired into 140 cases of fire, and has made a personal investigation on the spot of many of these. It is worth while to know that in 13 cases fires were directly due to defective electrical arrangements—a point which institutional workers should always bear in mind. Dr. Waldo strongly urges that jurisdiction given to the City Coroner under the City of London Fire Inquests Act of 1884 should, in the public interest, be extended throughout the country to all coroners, and that the Act, in fact, should be made compulsory. With that recommendation everyone who is aware of the exceeding usefulness of such fire inquiries as the City Coroner holds will cordially agree.

